# Aesthetic Analysis of the Regular Style of Sichuan Potted Landscapes in China

**DOI:** 10.3390/plants11202784

**Published:** 2022-10-20

**Authors:** Yanling Hao, Shixun Hu, Hai Xiao, Shiliang Liu

**Affiliations:** 1 Department of Agronomy & Horticulture, Chengdu Agricultural College, Chengdu 611130, China; 2 College of Landscape Architecture, Sichuan Agricultural University, Chengdu 611130, China; 3 Chengdu Sanyi Gardening Engineering Co., Ltd., Chengdu 611130, China; 4 Sichuan Yuze (Xscape) Landscape Planning and Design Co., Ltd., Chengdu 610093, China

**Keywords:** Sichuan potted landscapes, regular-style potted landscapes, trunk shape, branch types, aesthetic analysis

## Abstract

Understanding the Sichuan potted landscapes, one of the five types of traditional Chinese potted landscapes, is important for exploring the development of Sichuan landscape architecture in Southwest China. In this study, we analyzed the shape and aesthetic characteristics of the traditional regular bonsai of the Sichuan style according to trunk features, branch patterns, tree shape, branch arrangement, and branch and plate numbers. Studies have shown that the flat-branch-type branch plate slopes downward and the bottom rises, and the number of branch plates is 2n + 1 (where n is the number of layers of the branch plate, n > 4), which is a proportional sequence. The ratio of the main pile height to the footplate is approximately 1.5:1–2:1, the pot:footplate ratio is <1:1, and the maximum bend:trunk ratio is <1:2. There were extremely significant differences between the length of the foot plate and its adjacent branch and plate length and the distance between the branch and plate. Interestingly, the rolling branch-type plants are characterized by vertical curved branches, oblique curved branches, and curved branches, reflecting the beauty of harmony and symmetry. Overall, the regular-style Sichuan tree potted landscape is mainly characterized by the beauty of rhythm and symmetry.

## 1. Introduction

Chengdu, Sichuan, is known as the land of abundance. The long cultural and historical evolution in the region has nurtured the development of the elegant and elaborate Bashu culture and rich and colorful local traditional arts, including the Sichuan art of potted landscapes (Bonsai and Penjing) that represents the intersection between landscape and culture. The Sichuan art of potted landscapes originated in the Shu-Han period, and the most prominent examples are the Zhangsong ginkgo potted landscapes in Lidui Park in Dujiangyan [1]. During the Tang and Song Dynasties, the production and modeling techniques of Sichuan potted landscapes with tree stumps gradually developed, and during the Ming and Qing Dynasties, the art form flourished. Many different shapes have been utilized: three-bend and nine-turn, regular-turn, upright, oblique, horizontal, and other styles [2].

Traditional Sichuan potted tree landscapes can be divided into two types: regular style and natural style. In the regular style, the branches and twigs of the entire plant are incorporated. Twigs are colloquially called lamellar branches [3], also known as branch discs [2], and each lamellar branch is unique. There are primarily three types of branches in regular-style tree potted landscapes: the flat branch type, rolling branch type, and semiflat/semirolled type [4]. The flat branch type can be further divided into two subtypes: the standard branch type and the nonstandard branch type. There are five formulas [5,6]. For the rolling branch type, there are specific rules regarding the internal structure of the branches, as well as the arrangement of the main branch and side branches. The rolling branch type can be further divided into two rhythmic forms: large rolling branches and small rolling branches [7,8].

The Sichuan-style regular tree stump bonsai model has existed in China for nearly a thousand years. Previous studies have retained a large number of documents, but some documents are difficult to understand due to their long history. At present, the existing modern literature on the shape of the branches and plates of Sichuan-style bonsai mainly describes ten regular bonsai and the Three Shapes and Five Styles modeling characteristics of their branches and plates [8,9]. In terms of academic classification, the classification methods of Sichuan-style regular bonsai are not the same, and there is no systematic and scientific analysis of its structural characteristics and aesthetic characteristics. At present, most scholars mainly use the 10 methods of Masters Sifu Chen [5] and Chuanrui Pan [3,4,5,6,7,8,9,10,11] to classify the trunk shape. Among them, except for Pan’s trunk shape, the dragon body and the pimple body, which are quite different from Chen’s method of connecting, bending, turning, and borrowing, the methods have different names but involve similar techniques. The difference is that the Dujiangyan Bonsai Association of Chengdu [10] records that the main plate bend of the Sichuan-style bonsai mainly includes four types: the opposite bend, the Han Wen bend, the three-drop bend, and the rolling dragon bend. In the Chengdu Bonsai document published in 1957 [9], the trunk shape of Sichuan-style bonsai is divided into more than 20 techniques, the first 10 of which are the same as Chen’s regular bonsai classification. In addition, Laichun Tang compiled 10 regular trunk techniques in the Bonsai Techniques [12,13,14,15]. There is only one trunk shape, Moonbow Dropping, which is slightly different from Chen’s classification, and the rest are all the same. The paper “Chongqing Regular Pile Head Bonsai Techniques”, by Taibin Wan and Pei Ma [16], pointed out that there are no branches in the bend of the main trunk, and when the arches are formed incorrectly, the branches must be flat branches, the branches must be dried, and the layers must be clear.

In general, there are many regular bonsai works with a history of hundreds or even thousands of years existing around Chengdu that are worthy of esoteric exploration. At the same time, the research on the shape of the branches and plates of the Sichuan-style bonsai mainly focuses on various branch and plate techniques, and there are few records of the arrangement of the relevant branches. To address this gap, this study analyzes the expressiveness, rhythm, stem proportion, and flexibility of the regular-style Sichuan potted tree landscape from the aspects of the trunk height, branch and plate shapes, quantity, arrangement, and branch and trunk ratio of the entire regular-stump bonsai. Its aesthetic patterns and forms are detailed so that the world can more easily understand the Sichuan-style bonsai and innovatively absorb and apply it to bonsai production and garden landscaping.

## 2. Results

### 2.1. Trunk Features of Tree Stumps of Regular-Style Sichuan Potted Landscapes

#### 2.1.1. Characteristics of Trunk Shape

**Figure 1:**
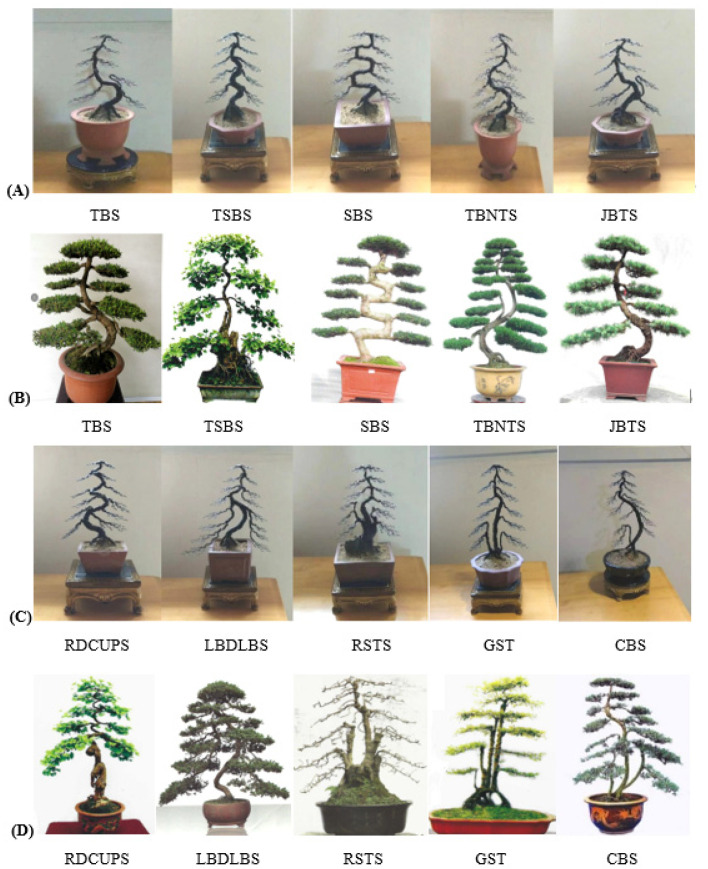
The standard models (**A**,**C**) and plant shapes (**B**,**D**) of the Sichuan regular potted landscapes. Note: The above images depict the ten techniques of regular-style Sichuan potted landscapes, including the Turn and Bend Skill (TBS, Chinese name ‘掉拐法’), the Turning Symmetrical Bend Skill (TSBS, Chinese name ‘对拐法’), the Square Bend Skill (SBS, Chinese name ‘方拐法’), the Three Bends and Nine Turn Skill (TBNTS, Chinese name ‘三弯九拐法’), the Joining the Bending and Turning Skill (JBTS, Chinese name ‘接弯掉拐法’), the Rolling Dragon Coil Up the Upright Post Skill (RDCUPS, Chinese name ‘滚龙抱柱法’), the Large Bends and Drooping Longer Branches Skill (LBDLBS, Chinese name ‘大弯垂枝法’), the Root Stump to the Top Skill (RSTS, Chinese name ‘逗身照篼法’), the Graft Straight Trunk Skill (GSTS, Chinese name ‘直身逗顶法’), and the Cleverly Borrowing Skill (CBS, Chinese name ‘巧借法’). The same applies below.

As shown in Figure 1, the traditional regular-style stump potted landscapes were characterized by a balanced tree, complete structure, elegant posture, regular form and adherence to strict rules. In the internal structure, the branches and stems were extremely tortuous and conformed to certain rules: the top and the root were nearly in a straight line and formed a central axis. All bends, whether symmetrical S bends or three-dimensional spiral bends, and the various forms of the stand-up and straight-up methods were symmetrical or rotated around the central axis, and the center of gravity of all trees was located on the centerline, which follows the general law of plant growth in nature [17,18]. Moreover, each layer of the tree crown was composed of two branches, one left and one right, which were symmetrically distributed on the left and right sides of the main trunk [18,19]. The front and rear sides of the main trunk had no branches, so the orderly relationship between the trunk and the branches was not hidden but fully revealed.

**Figure 2:**
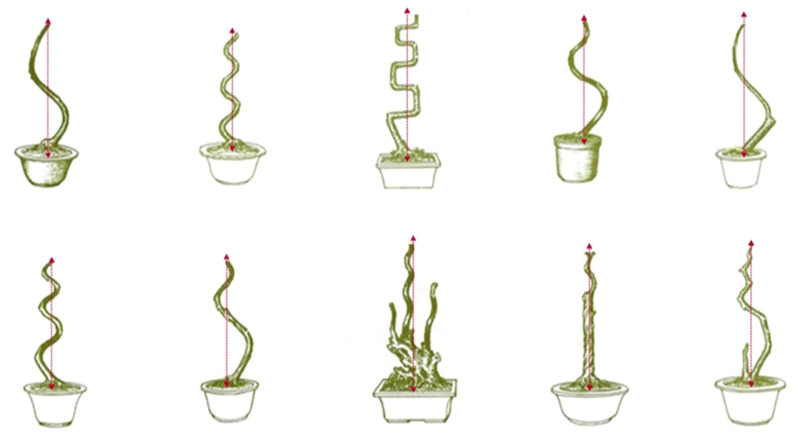
The position of the central axis of regular-style Sichuan potted landscapes.

We found that the center of gravity of all trees was located on the centerline, which was also in accordance with the general law of natural plant growth. Moreover, each layer consisted of left and right branches, which were symmetrically distributed on both sides of the trunk [1,13]. There were no branches on the front or back sides of the trunk, and the systematic relationship between the trunk and the branches was completely exposed. The system was prominent, and artistic features were naturally formed (Figure 2). Overall, the closed pattern formed by the left and right branchlets of the whole plant formed diagonal lines, and the lengths of the branchlets at the bottom and top were equilateral triangles (Table 1).

#### 2.1.2. Front/Side Expressions of Stumps

**Figure 3:**
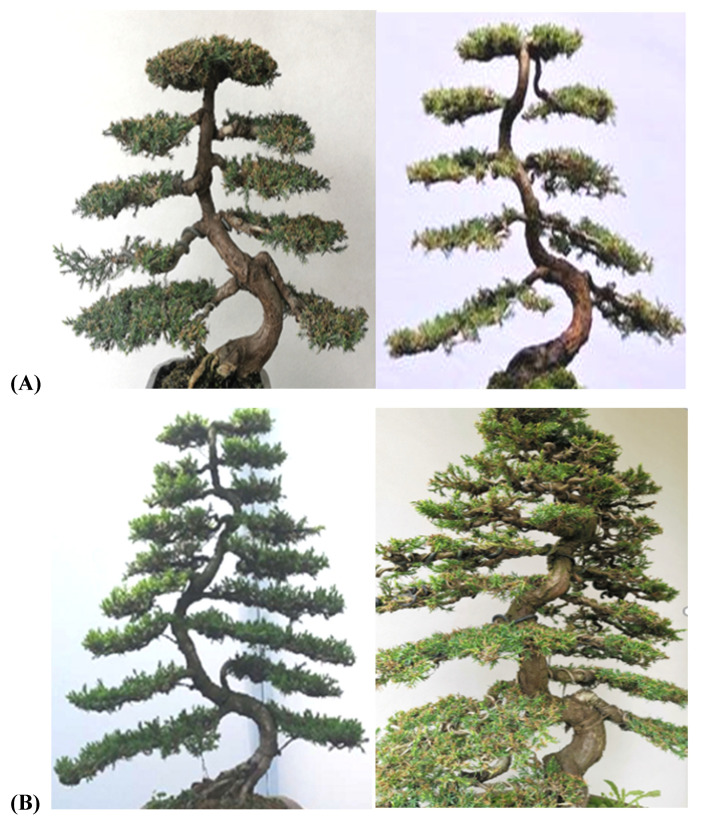

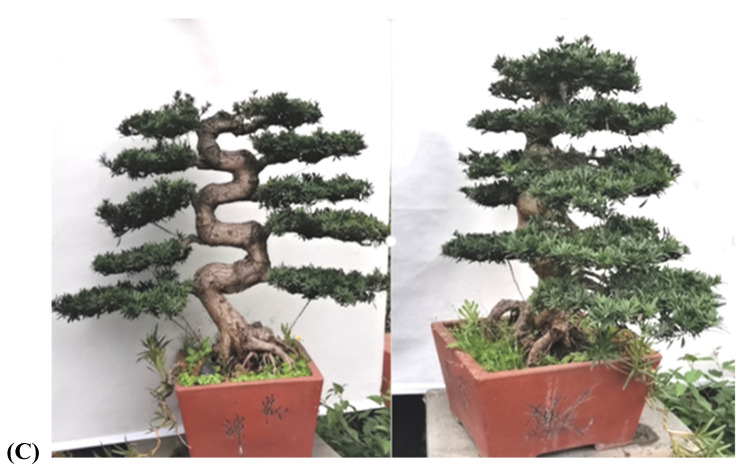
Front/side views of the TBS (**A**), TBNTS (**B**), and SBS (**C**) methods.

As seen in Figure 3, the regular-style Sichuan potted landscapes had a strong sense of rhythm, which was mainly reflected in the shape of the trunk; this is commonly known as the body method. All regular-style potted landscapes have their own rhythmic tones, among which the drop-turn (Diaoguai) method, the three-bend and nine-turn (Sanwanjiuguai) method, and the regular-turn (Fangguai) method are the most prominent [14]. Usually, when artists create bends, the first bend is made toward the artist in the front, and then, the directions of the bends alternate. Interestingly, in the drop-turn (Diaoguai) method, when viewed from the front, the curved shape of the entire plant must conform to the shape specified by the saying “One Bend is Large, The Second Bend is Small, The Third Bend and Fourth Bends Cannot Be Seen”. The three-bend and nine-turn (Sanwanjiuguai) method has three large bends when viewed from the front and nine large bends when viewed from the side [20,21]. The method of regular turning (Fangguai) follows the standard of Chinese crutches on the front and a line on the side. The above rules form the unique artistic characteristics of Sichuan potted landscapes. The rhythm is apparent, so it is known worldwide as rhythmic poetry.

#### 2.1.3. Proportion of Branches and Trunks

Traditional regular-style Sichuan potted landscapes are characterized by the proportional relationship among the branches. The length of the branch plate on the stump is generally determined by the length of the foot plate (from the base of the trunk, the first branch plate) [22,23]. The length of the foot plate is usually in proportion to the rest of the stump potted landscapes. The ratio between the length of the foot plate and the diameter of the pot is usually between 1.5:1 and 2:1, and the ratio between the overall length of the foot plate and the height of the stump should be between 1.5:1 and 2:1 or 1:1 (meaning the maximum length of the foot plate should not be greater than the height of the stump) (Table 2).

In this study, we also found that there were some customary ratios for the relative heights of various parts of the trunk. For example, there was a large bend in the lower part of the trunk for the drop-turn (Figure 3A), three-bend nine-turn (Figure 3B), and regular-turn (Figure 3C) methods. The customary ratio between the height of this large bend and the height of the entire stump was 1:2; that is, the height of the large bend was approximately half of the entire height. These processing ratios help the branches of regular-style potted landscapes achieve the artistic effects of symmetrical shape and harmonious proportions.

### 2.2. Characteristics of the Flat Branch Method of the Sichuan Potted Landscapes

#### 2.2.1. Expression of the Flat Branch Style

**Figure 4:**
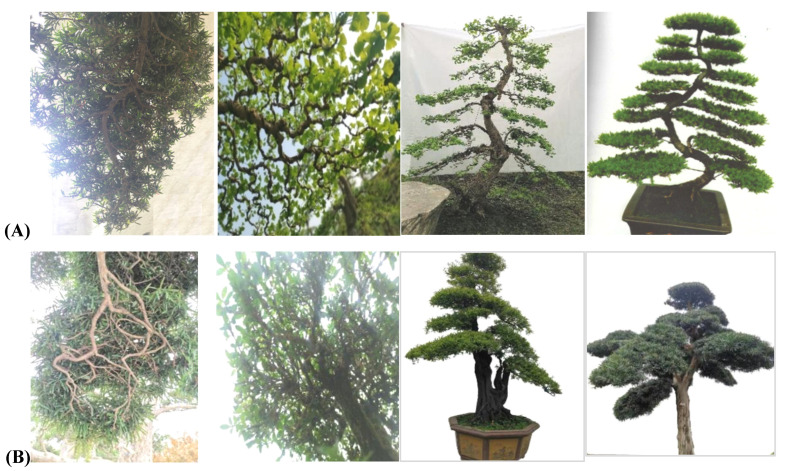
The branch styles of standard (**A**) and nonstandard (**B**) flat-branched types.

There are two types of traditional flat-branched trees in Sichuan potted landscapes: standard flat-branched and nonstandard flat-branched types. The common features of the two types are that the twisted and flat branches of the entire plant are flat and downward sloping, and the leaf tips are raised. However, there are obvious differences between the two types of branches; standard flat-branched branches have clear veins, some left branchlets that are similar to leaves, and some right branchlets that are similar to lateral veins, which are evenly distributed on both sides of the main branch, and all branches are neatly arranged [17,18]. In contrast, nonstandard flat-branched branches are relatively free, the left and right side branches are unclear, and the side branches can be on the right or left and point up or down, but, regardless, they fill in the branches to make them plump and natural (Figure 4).

#### 2.2.2. Number and Length of Branch Plates

Traditional regular-style Sichuan tree potted landscapes have 5–15 layers of branches from bottom to top; the top layer usually has only one branch, which is formed by the confluence of left and right branches, and the numbers of branches and twigs of the whole tree are consistent [19,20]. The number of branches followed the geometric series 2n + 1 (n is the number of branch layers; the branches from left to right are referred to as a layer; n > 4), and there was an odd number of branches. The probability of branching (PBB) of all trunk shapes was greater than 80%, and the probability of TSBS and SBS returning to generate branches was as high as 100%. In terms of the probability that the left and right branches were generated, that of TS was also greater than 80%, and those of TSBS, SBS and RDCUPS were as high as 100%. All the branches of TBS, TSBS, SBS, and GBTS had five layers with approximately nine branches; RSGTS and GSTS had between 5 and 7 bends and 9 and 11 branches in common, respectively; TBNSTS and LBDLBS had 9 layers of branches with 3 times the number of branches: 3, 6, 9, and 12 branches. However, there was no obvious pattern for RDCUPS and CBS, which may be due to the indeterminate number of winding loops for RDCUPS and the uncertainty of combinations for CBS (Table 3).

Table 4 shows that the ratio of foot branch length to adjacent branch length and the distance ratio of all regular-style potted landscapes from bottom to top, including BLSF (branch length from left to right of foundation pile) and branch distance (the first branch to the distance between the foundation piles, abbreviated as BD), varied significantly [21,24]. There were significant differences in the length ratio and distance ratio of the first three branches, with the length decreasing and the distance decreasing sequentially. The length ratios and distance ratios of all the branches decreased sequentially, so the lengths and distances of all the branches also decreased sequentially, but the difference in the correlation ratios was not significant.

#### 2.2.3. Arrangement of Branches on the Trunk

**Figure 5:**
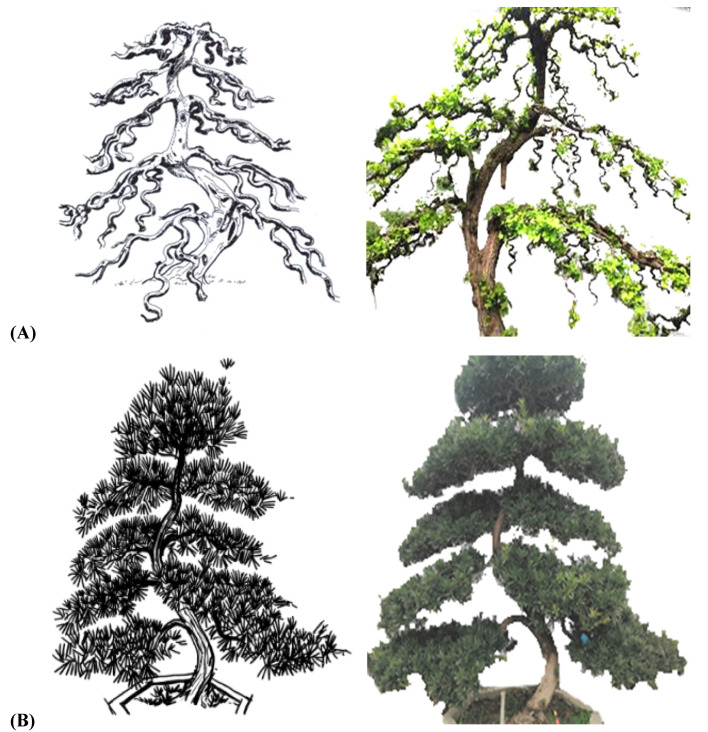
Comparison of the standard (**A**) and nonstandard (**B**) flat-branch styles.

The branches in the traditional regular-style Sichuan tree potted landscape imitate the natural shape of the natural tree crown, so all potted landscape artists shape the branches into structured layers. Each layer consists of a pair of branches, and several branches are arranged according to the rules of the structure to form a regular canopy [24,25]. In the canopy, all branches were arranged symmetrically on the left and right sides of the trunk. The branches were all distributed on the back of the curved trunk so that the veins between the trunk and the branches were completely exposed, exhibiting a high degree of organization. Studies have shown that the relationship between the main branch and the side branches is as follows: the main branch is in the middle, and the side branches are arranged on the sides of the main branch and look similar to pinnate veins (Figure 5).

#### 2.2.4. Metric Analysis of Flat-Branched Bonsai

**Figure 6:**
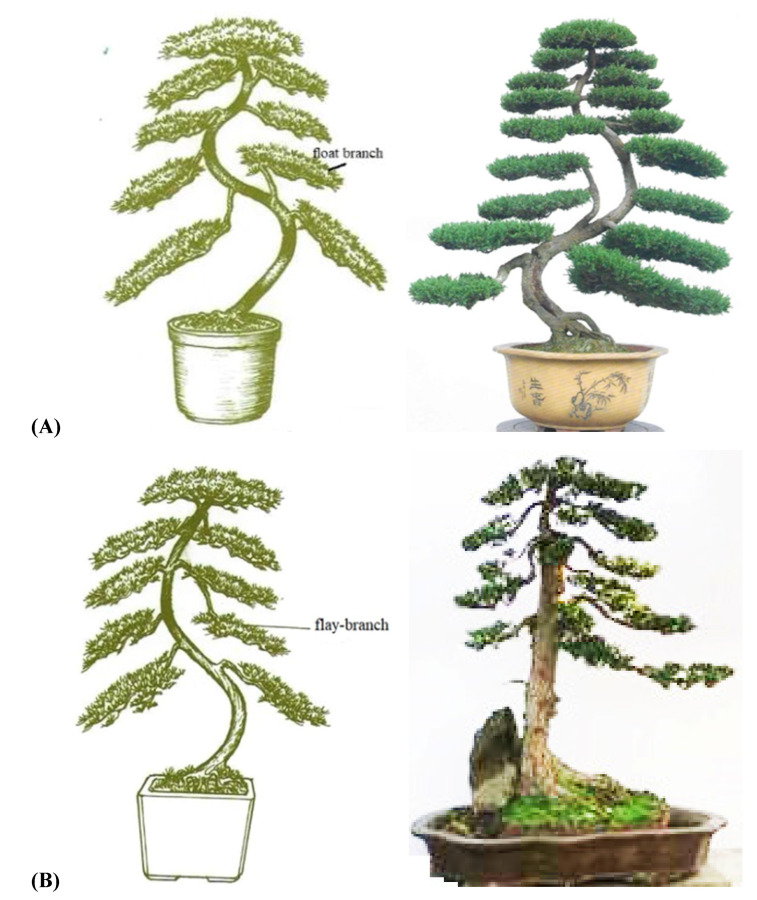
The traditional float (**A**) and flay (**B**) branch styles.

In production, it is not easy to achieve a standard regular pattern. The tree stumps in potted landscapes have natural forms. Due to the limited number of branches of tree stumps, branch trays are sometimes not grown in predetermined positions but are grown to preserve the symmetry of the trunk and balance. However, flay and float branches can overcome this issue because of their flexibility (Figure 6). The flame branch approach involves taking one or two branches from the branches and layers above the same trunk, and if the float branch approach is utilized, one or two branches are also drawn from the lower branches and layers of the same trunk. The presence of flay and float branches follows production guidelines, which include rich changes, order, and flexibility, and can be used not only to meet the aesthetic requirements but also shorten the production cycle.

### 2.3. The Characteristics of the Rolling Branches in Sichuan Potted Landscapes

#### 2.3.1. Expression Form of the Rolling Branch

**Figure 7:**
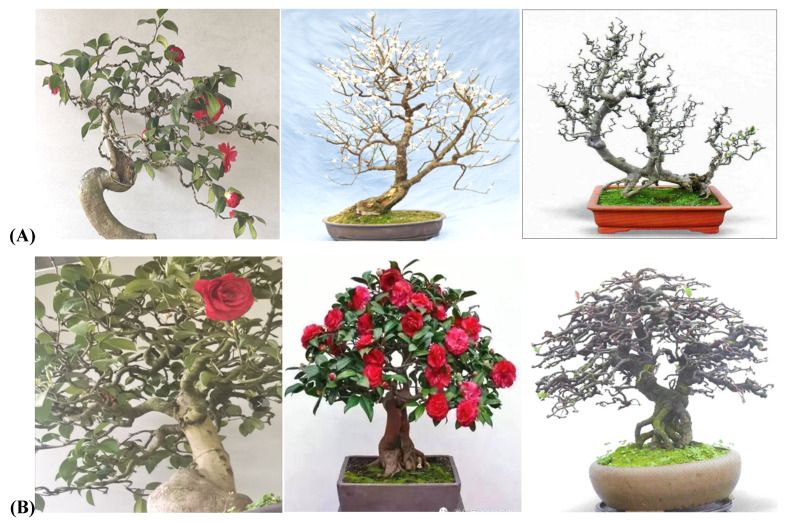
The small (**A**) and large (**B**) rolling-branch styles.

As shown in Figure 7, compared with the copolymerization and strict requirements of the flat branch type, there were no strict requirements for the stump branches in the rolling branch-type potted landscapes, but they must contribute to the complete and harmonious aesthetics of the entire plant [25,26,27]. In other words, the aims of branching are for each branch to be harmonious and natural, the curves to have their own characteristics, and the branches of the entire plant to be elegant and full-bodied. However, certain differences were observed: small curly branches were evenly arranged and scattered randomly, and for the large curly branches, the leaves and flowers were spaced evenly and faced outward.

#### 2.3.2. Styling Features of the Rolling Branch

From the lateral view, both the large curly branch type and the small curly branch type had a regular cone shape surrounding the trunk, and the branches were distributed in a well-proportioned manner throughout the stump. From the vertical perspective, however, both were elliptical. All branches were neatly arranged around the trunk, and the space among the branches was full and natural.

**Figure 8:**
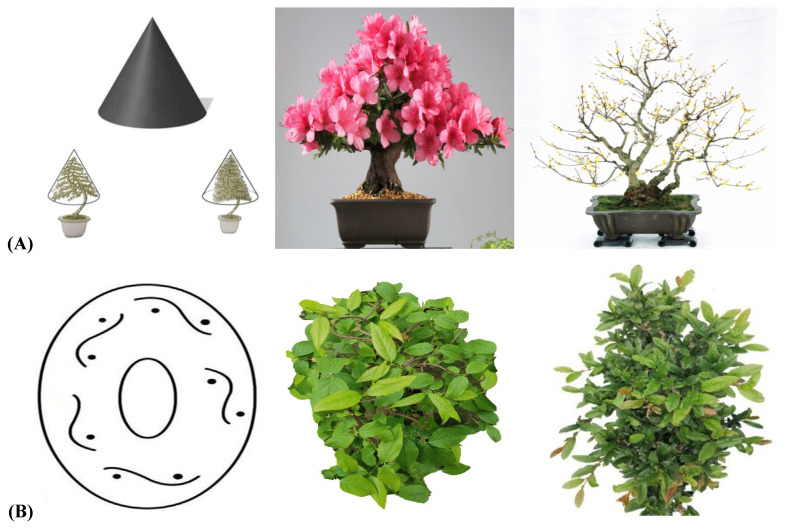
The horizontal (**A**) and frontal (**B**) views of the large/small rolling-branch style.

For large branches, the following features were observed: the leaves and branches faced outward slightly; the top of the trunk, side branches, and branches were not cut off; all the space was filled; the leaves faced outward; the top of the branches faced outward; and the entire tree potted landscapes were plump and formed a standard cone, arranged neatly, with no convexity or concavity. For small branches, the top of the trunk did not stick, but there were vertical bent branches, oblique bent branches, and back bent branches. There were gaps in the branches, branches were pointed at open areas, the branches of the entire plant were evenly arranged, and the overall form was cone-shaped [26,27]. For half-flat- and half-rolling-branch types, the model branches looked similar to the petals of a Golden Chrysanthemum; each petal had its own direction, and petals of the same lengths formed a plane. At the initial stage, the branches were sparse, and the power of branching was weak; some were flat, and some were rolling. At the later stage, the number of branches increased and evolved to form a flat branch (Figure 8).

#### 2.3.3. Metric Analysis of the Rolling Branch

**Figure 9:**
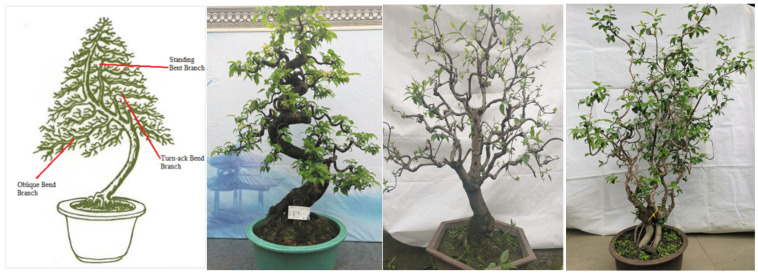
Three branch-making styles (SBB, OBB, and TBB) for the rolling-branch bonsai. Note: SBB: Standing Bent Branch (Chinese name ‘立弯枝’); OBB: Oblique Bent Branch (Chinese name ‘斜弯枝’); TBB: Turn-back Bent Branch (Chinese name ‘回曲枝’).

For Sichuan potted landscapes, if the flat branch type highlights the overall effect, then the curly branch type is more prominent in terms of the combination of flowers and leaves [3,5,7]. In the flowering season, the flower branches show the beauty of the flowers, and each flower is perfectly displayed. The flowers are evenly spaced. After the flowers fall, the leaves fill the entire space, and the leaves face outward and upward. For the space to appear full and the branches natural, it is necessary to thoughtfully and flexibly apply vertical, oblique, and twisted branches in potted landscapes with curly branches. Research has shown that vertical curved branches are generally used to fill the main frame space. Oblique curved branches are curved branches with a certain inclination and were used to fill the angular space; meandering branches were used to fill the space between upper and lower branches or between left and right branches (Figure 9).

### 2.4. The Characteristics of the Semiflat/Semirolled Branches

**Figure 10:**
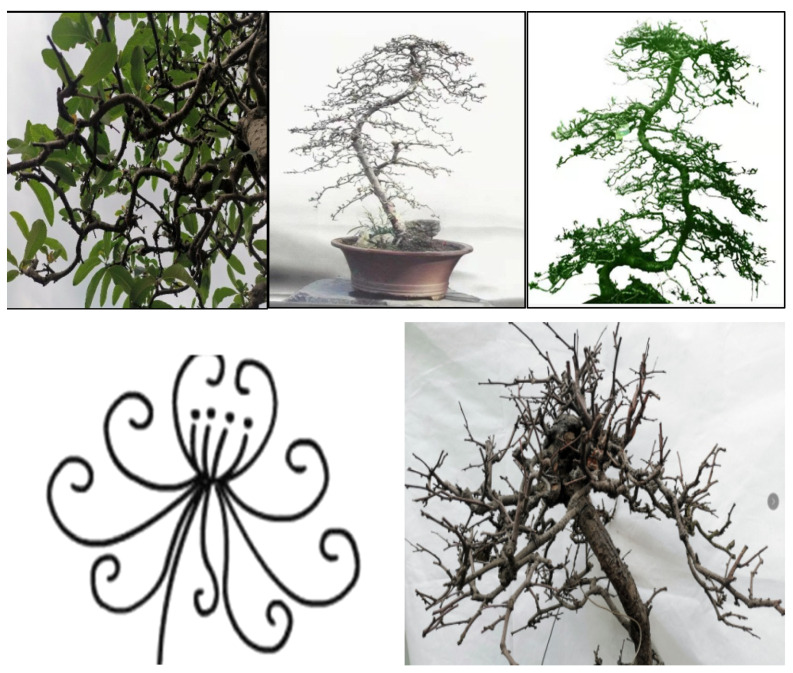
Styling features of the semiflat/semirolled branches.

The semiflat/semirolled type is between the flat branch and the rolling branch types. The flower branch approach was similar to that of the flat branch type but also similar to the small rolling branch subtype of the rolling branch type. The only difference was that there were no twisted branches and vertical curved branches with several consecutive curves or occasional curved branches (Figure 10).

It takes many years to add one or two layers of branches to the pile head for the flat branch type. Due to the strong growth potential of the tree species, the pile head of this style increases annually, and the branch plate transitions to a flat branch type with the increase, forming a flat branch-type pile head. The switch to a flat branch-type pile head was determined by the development of the tree species and the choice of timing to fill in the scorpion, determining when some branches should be added or cut off. It was easier to convert *Malus halliana* to the flat-branched type and slightly more difficult to convert *Chaenomeles speciosa*. Most of the pile heads of this style had no branches on the top and had pointed tops when they were first plastered. Therefore, the ancient *M. halliana* and the stem heads of *C. speciosa* all exhibited a flat branch-type plate with a dignified and vigorous posture (Figure 10). Tree species with both leaves and flowers that retain their leaves after flowering were suitable for semiflat/semirolled tying and included *M. halliana*, *M. micromalus*, *C. speciosa, C. cathayensis*, and *M. spectabilis* (Figure 10).

## 3. Discussion

### 3.1. The Branches/Panels of the Modeling Tree Serve Completely around the Main Trunk

In this study, we analyze the overall aesthetic characteristics of regular-style Sichuan tree potted landscapes from the aspects of the expression of form, rhythm analysis, branch ratio, rhythm performance, and art form and technique. The results showed that all the branches were extremely tortuous, following the principles of modern mechanics and geometry. The spatial changes in trunks were rhythmic, the proportions of branches were coordinated and orderly, and the changes in rhythm reflected a certain flexibility.

Our work mainly investigates the trunk of Sichuan-style regular bonsai and focuses on its modeling characteristics. The results showed that all the branches and plates serve completely around the trunk, with completely different fronts and sides, which is also a way of balancing the layout of the trunk. The method of tying the branches and the method of the trunk should complement each other to form a complete regular tree stump. Judging from the use of ancient documents and contemporary bonsai works, most of the TBS, TSBS, and JBTS exist in pairs. The common tree species are *Ginkgo biloba* and conifers. This type of bonsai is generally placed on both sides of the main roads of shops, entrance gates, large and medium-sized enterprises, and on both sides of the door of the home and the alley, as majestic as a door god and general. However, the TBNTS, LBDLBS, and RDCUPS bonsai are mostly placed in large squares, temples, and other solemn public places, with arhat and juniper being the most common. In addition, other bonsais, such as CBS, GSTS, and RSTS, are mostly species such as *D. cathayensis*, *S. japonica*, and *L. indica*. These bonsais have unique shapes and are placed in different positions [27,28,29].

### 3.2. Rigorous Rhythm Is the Basic Structural Principle of Art Form in Sichuan Bonsai

Judging from the ancient documents recorded in the past dynasties and the actual measurement results of the existing regular bonsai branches, there are three methods for the traditional Sichuan-style bonsai branches, namely, the flat branch type, the rolling branch type, and the semiflat and semirolled type. Among them, the flat branch type is divided into two types, the standard type and the flower branch type, and the two have different regular practices in forming the internal structure of the branch plate and the arrangement relationship of the main branch and the side branches. Each curved back of the trunk has a branch plate, which is symmetrical from left to right. The vertical distance between the branches and plates gradually decreases. There is a certain proportion of coordination between the branch plate and the pot, the foot plate, and the height of the plant. Combined with the shape of the whole plant, this technique embodies symmetrical beauty and grammatical beauty. The branches are fan-shaped and sloping downward, and the ends of the branches are slightly raised. According to records, this style imitates the stalwart temperament of the branches that are weighted down by the perennial snow in the Minshan Mountains [30].

Rigorous rhythm is the basic structural principle of the art form; the prominent characteristics were rhythmic beauty and symmetry, and the samples conformed to artistic aesthetics and general art principles [31]. The types of flat branches were obviously different and needed to be determined to maintain harmony with the trunk and the branches; the branches and trunk must be visible. The layers were distinct, and the branches changed from drooping to flat or slightly inclined [32]. The branch ends were slightly raised, and the collateral veins were clear, which may be due to the lack of sunshine in Sichuan [33]. Furthermore, the number of branches on the trunk was different; all the branches were at the top and grew over time. Sometimes, they were counted as one branch, but they were counted as two branches at other times, depending on the ancient ideals and traditions [34,35]. In addition, almost all the branches of the regular-style Sichuan tree potted landscapes changed rhythmically from left to right. The lengths of the branches and the distances between the branches started to decrease from the bottom of the stump, and the curvilinear trunk exhibited constantly alternating left and right changes, so that the entire tree potted landscape was endowed with a dynamic and structured sense of vitality; this ornamental beauty and delicacy of the stump potted landscape morphology is intended to give people the strength and inspiration [36].

The symmetry, balance, and rhythm of Sichuan-style bonsai have deeper meanings and connotations. In the traditional aesthetic structure of the Chinese nation, the spirit of rationality permeates all structural levels and is manifested in the poems and songs of the literati of the past dynasties, not only to express the mind of the literati and the writer but also to reflect the overall rhythm and rhyme. The traditional aesthetic has a vast artistic and broad cultural heritage [26]. In considering aesthetic objects, Chinese people tend to appreciate rationality. For works of art, they tend to seek logical explanations, and they entrust the artwork with their own wishes and good wishes [33]. This kind of aesthetic psychological characteristic also existed in the Chengdu bonsai artists in the past dynasties and is strongly reflected in the bonsai art activities. 

The Sichuan-style regular tree stump bonsai clearly embodies the spirit of legality, ethics, logical morality, and so on. The number of branches and plates on a bonsai, in ancient times, reflected these values; for example, 5–6 layers represent the wishes for good fortune and are used by ordinary people; 7–8 layers are used by officials, representing their prosperity; and 9–12 layers are used by emperors and relatives of the country, representing the dignity of identity. This symbolism is integrated with the natural scene reflected by the bonsai, and the natural landscape is extracted from a primitive, simple, scattered, and purposeless state and condensed into a fully embodied Bashu cultural tradition and rational principles, with trees as the perfectly reproduced carrier [32].

### 3.3. The Harmonious Presence of Selected Plant Tissues Is Reflected in Sichuan Bonsai

Many flower and fruit plant materials have been used in Sichuan potted landscapes. The plant pots reflected the harmonious existence of flowers and leaves. The rolling branch was a unique characteristic technique of Sichuan potted landscapes [9]. Flower and fruit potted landscapes exhibited the harmony of flowers and leaves. In general, to highlight the beauty of these plants when blooming or bearing fruit, the location of the different tree species, the location of the flower buds, the distribution of branches when the flowers were in full bloom, and the overall effect of the leaves and fruits after the flowers fell were different. The branching angle has important ornamental value for potted camellia landscapes and potted rhododendron landscapes [14,28]. The camellias are larger, and the leaves are lush, accentuating the full tree when it bloomed, as well as the branches and leaves when the flowers fell. Studies have shown that branch curling overcomes many problems, and this technique achieves compact and natural beauty through the logical arrangement of branches. Similarly, the semiflat/semirolled branching method was very effective for tree species with weak branching power. All branches were evenly distributed within the same layer to achieve full branches that were elegant, natural, intact, and beautiful. Evergreen broad-leaved tree species or top-flowering tree species were suitable for the large, curly branch type of potted landscapes, including rhododendron, camellia, and osmanthus [29,36]. For the small, curly branch type, trees with flowers and leaves that bloomed at the same time and trees with long leaves after flowering were more suitable, including plums, cherry blossoms, plum blossoms, and other plants with flowers in the leaf axils. For the semiflat/semirolled types, plants such as begonia, papaya, and pygmy begonia with flowers and leaves that open at the same time were used.

For example, the Sichuan-style rolling bonsai is divided into two metrical forms, the large rolling branch type and the small rolling branch type. The two are similar to the semiflat and half-rolled branch methods, but there are many differences between them. The stump bonsai of the two techniques still has a vertical central axis at the tip and base of the stump. The rolling branch bonsai is dominated by the central axis to form a positive cone, which is mainly seen in Sichuan flower and fruit bonsai. Bonsai artists can determine the type of techniques used according to the sizes of the flowers, fruits and leaves, such as *Camellia*-, *Rhododendron*-, and *Malus*-type bonsai with large flowers and fruits. To ensure that there is no interaction between flowers, fruits, and leaves, the overall performance is uniform and beautiful. The use of large rolling branches can ensure that the flowers and fruits of each branch are evenly exposed, and the flowers, leaves, and fruits behave naturally and smoothly. The flowers and fruits of plants such as *Chimonanthus praecox*, *Prunus mume*, and *Malus halliana* are relatively small. Using the small rolling method can effectively display the effect of flowery branches. For plants similar to begonia, with few natural branches and a certain curvature, the semiflat and semirolled branch method is most suitable. Most of the existing bonsai works with sticking stems are made by this method. The central axis formed by the main trunk becomes the balance point of left and right symmetry. All single branches start as flat branches and turn into rolled branches at the end. However, the single branches on all sides are basically on the same plane. The trunk shape is generally based on the method of turning off and turning on, and the branches change from flat to rolling, which is reflected in the beauty of symmetry and harmony as a whole [33,34,35,36,37].

## 4. Methods

Sichuan potted tree landscapes are mainly found in Chengdu and are, therefore, also known as Chengdu potted landscapes. More specifically, they are mainly distributed in Qingyang, Jinjiang, Wenjiang, Dujiangyan, Pidu, Shuangliu, Xindu, Dayi, and Pujiang, Chengdu, Sichuan. The art form referred to as the East Sichuan School can be found in the Nan’an, Beibei, Wanzhou and other areas surrounding Chongqing, China.

Taking Sichuan bonsai as an example, this study focused on visiting 10 Sichuan-style bonsai museums, including the bonsai garden of Chengdu Dufu Thatched Cottage Museum, the bonsai garden of Chengdu Wuhou Temple Museum, the bonsai garden of Chengdu Baihuatan Park, the bonsai garden of Chengdu People’s Park, the Lidui Park of Dujiangyan, the bonsai museum of Sanyi Horticulture, the Chengdu Wangjianglou Park, Chengdu Wenjiang Chen’s bonsai garden (Kaiqin Chen’s former residence), Baihua Garden in Zigong Guangzhou Garden, and Chongqing Nanshan Botanical Garden bonsai garden, as well as 108 bonsai gardens in 9 municipal districts, 4 county-level cities, and 6 counties around Chengdu and 32 bonsai gardens in Chongqing, including Xiaochangkou, Guanyin Bridge, Daping, Dadukou, and Yangjiaping. The research samples were determined in accordance with the classification method and related standards of Chen’s Banjing Pile-Heading Technique [5], and the number of samples exceeded 5000 (60% of the test samples were from Chengdu, and 40% of the test samples were from Chongqing). These sample materials are mainly local tree species in Sichuan, including *Ginkgo biloba*, *Diospyros cathayensis*, *Serissa japonica*, *Podocarpus macrophyllus*, *Prunus mume*, *Rhododendron simsii*, *Malus spectabilis*, *Lagerstroemia indica*, etc., referring to the famous bonsai literature and questionnaires in ancient history (traditional regular bonsai production technical points), as well as field work observations, explanations from 100 bonsai masters (belonging to generations of bonsai family inheritors) during on-site production, and accurate measurement methods.

In particular, several research methods were utilized: field visits, questionnaires, interviews with well-known potted landscape artists, comparisons of potted landscape books, the observation of field works in various regions, obtaining on-site production explanations by potted landscape masters, and comparisons of product quality. Different types of data were collected during the study: plant height, number of branches and trays, distance between branches and trays, length of branches and trays (the distance from the base of the branch to the center of the tip), the ratio of the length of the branches, and the height and width of the pot where the stump was located. More than 5000 potted landscapes were tested. The classification of potted landscape samples was performed in accordance with the classification method and related standards of Chen’s “The potted landscapes Pile-head Pantying Technique” [5]. There are ten techniques for the production Sichuan potted landscapes: the Turn and Bend Skill (TBS), the Turning Symmetrical Bend Skill (TSBS), the regular Bend Skill (SBS), the Three Bends and Nine Turn Skill (TBNTS), the Joining the Bending and Turning Skill (JBTS), the Rolling Dragon Coil Up the Upright Post Skill (RDCUPS), the Large Bends and Drooping Longer Branches Skill (LBDLBS), the Root Stump to the Top Skill (RSTS), the Graft Straight Trunk Skill (GSTS), and the Cleverly Borrowing Skill (CBS) [14,15,16,17,22,23]. The test measurements were carried out with an international standard meter ruler, and the unit was cm (centimeters). Statistical analysis was performed using the SPSS 11.3 software (IBM Corp., Armonk, NY, USA), and analysis of variance and detection of statistically significant differences (0.05 level) were used.

## 5. Conclusions

In Sichuan regular-style bonsai, under the principle of following a strict structural rhythm, all the tissues of the modeled trees, such as branches, panels, flowers, and fruits, etc., are shown to exist harmoniously and serve completely around the main trunk, reflecting the ethical spirit and logical morality. Specifically, the trunks of ten kinds of bonsai with different shapes rotate in a straight line around the center formed by the top of the trunk and the root. The “flat branch-style” branch plate of the Sichuan school slopes downward, and the bottom rises. There are certain regularities between the number of branch plates, the height of the main pile and the foot plate, the pot and the foot plate, and the largest bend and the trunk. The front of the whole plant is trapezoidal in shape, and the sides are different, which embodies the beauty of rhythm, symmetry, and harmony. The rolling branch plant type of the Sichuan style is conical from the plane view and oval from the top view, emphasizing the harmonious beauty and symmetry of the plant. These findings of this study could evoke a deep understanding of the Sichuan regular-style bonsai, and on this basis, innovatively develop Sichuan-style bonsai while absorbing and applying it to Sichuan bonsai production and urban garden construction.

## Figures and Tables

**Table 1:** Overall modeling analysis of 10 shapes of regular-style Sichuan potted landscapes.

MTS		LBC (cm)	OLCL (cm)	OLCR (cm)	LTB (cm)
1	TBS	156.01 ± 36.8	135.12 ± 25.86	134.89 ± 10.22	91.02 ± 19.23
2	TSBS	197.01 ± 66.38	206.81 ± 14.35	208.01 ± 33.26	93.22 ± 15.65
3	SBS	148.26 ± 48.12	144.25 ± 33.66	143.01 ± 61.23	98.16 ± 26.35
4	TBNTS	190.23 ± 34.42	148.66 ± 21.12	149.02 ± 41.23	94.56 ± 31.55
5	JBTS	245.64 ± 58.23	131.45 ± 10.87	129.47 ± 33.65	155.31 ± 23.16
6	RDCUPS	200.15 ± 66.42	188.01 ± 41.17	191.34 ± 98.56	90.56 ± 18.97
7	LBDLBS	230.56 ± 41.20	195.23 ± 47.12	199.43 ± 56.23	120.56 ± 23.65
8	RSTS	225.61 ± 32.56	184.01 ± 33.21	182.66 ± 45.21	70.45 ± 9.85
9	GSTS	155.56 ± 22.45	299.99 ± 66.23	302.45 ± 36.25	88.6 ± 16.91
10	CBS	270.45 ± 58.14	267.21 ± 47.25	262.95 ± 33.56	80.99 ± 19.22

Note: MTS: Main Trunk Shape; LBC: Length of Basal Branchlets; OLCL: Oblique Length of Branchlets on the Left; OLCR: Oblique Length of Branchlets on the Right; LTB: Length of Topmost Branchlets. The statistics reported are the mean ± standard error (n ≥ 3).

**Table 2:** The average proportion of branches/stems of regular-style potted landscapes.

MTS		HMS/BB	BC/HMS	MBD/HMS
1	TBS	1.631:1	0.637:1	0.443:1
2	TSBS	1.668:1	0.684:1	0.286:1
3	SBS	1.583:1	0.964:1	0.213:1
4	RDCUPS	1.883:1	0.917:1	0.318:1
5	TBNTS	1.963:1	0.806:1	0.496:1
6	GSTS	1.885:1	0.751:1	0.457:1
7	LBDLBS	1.913:1	0.981:1	0.488:1
8	RSTS	1.743:1	0.714:1	0.112:1~0.428:1
9	JBTS	1.816:1	0.626:1	0.146:1
10	CBS	1.754:1	0.715:1	0.211:1~0.467:1

Note: HMS: Height of Main Stump; BB: Basin Bowl; MBD: Maximum Bend Diameter; BC: Basal Cladoceran; MTS: Main Trunk Shape. The values in front of the above ratio are the mean (n ≥ 3), which is calculated based on the measurement data of different plants of the same type.

**Table 3:** Comparison of the branch plates of the ten kinds of regular-style potted landscapes.

TS	NBTP	PN (%)	PBB/PBI (%)	LRP (%)
TBS	5L9B	92.31 ± 1.31	91.60 ± 1.23	92.60 ± 9.87
TSBS	5L9B	90.21 ± 1.23	100.00 ± 0.36	100.00 ± 0.99
SBS	5L9B	75.23 ± 3.36	100.00 ± 0.66	100.00 ± 0.65
TBNTS	7~9L1517B	79.77 ± 5.63	87.40 ± 6.35	88.70 ± 6.21
GBTS	5L9B	91.03 ± 0.63	93.10 ± 5.63	96.50 ± 9.15
RDCUPS	7~9L1517B	53.44 ± 6.66	93.50 ± 6.98	100.00 ± 0.36
LBDLBS	7~9L1517B	71.23 ± 5.63	87.50 ± 9.18	86.30 ± 9.12
RSGTS	5~7L9-15B	75.42 ± 6.69	86.50 ± 3.65	89.90 ± 4.56
GSTS	5~7L9-15B	68.74 ± 7.15	95.60 ± 6.69	86.70 ± 9.15
CBS	5~7L9-15B	48.76 ± 6.35	87.10 ± 6.98	89.50 ± 6.25
Mean	~7	74.61 ± 5.62	92.23 ± 7.13	93.02 ± 6.98

Note: PN: Probability of the Number; NBTP: Number of Branches on the Trunk Probability; B: Branch Plate; L: Layer. LRP, Left and Right Probability; PBB, Probability Bent Back; PBI, Probability Bent Inside. The statistics reported are the mean ± standard error (n ≥ 3).

**Table 4:** Length ratio of the adjacent branch plate.

TS	NB	RBASD	RABML
TBS	5	1.84 **:1.54 *:1.48:1.31	4.01 **:3.10 *:2.62:2.14:1.76
TSBS	5	1.58 **:1.30 *:1.28:1.16	4.42 **:3.61 *:3.01:2.58:2.11:1.72
SBS	5	1.54 **:1.16 *:0.88:0.72	2.11 **:1.69 *:1.35 *:1.04:0.85:0.68
TBNTS	9	1.55 **:1.19 *:0.99 *:0.86:0.79:0.73: 0.68:0.64	2.22 **:1.86 *:1.62 *:1.38:1.21:1.13:0.98: 0.79:0.66
JBTS	7	1.71 **:1.06 *:0.88 *:0.82:0.79:0.77	3.25 **:2.52 *:2.36 *:2.09:1.77:1.61:1.56
RDCUPS	9	1.51 **:1.25 *:1.17 *:1.11:0.96:0.91:0.88	3.61 **:2.66 *:2.41 *:2.22:2.09:1.88:1.71:1.61:1.54
LBDLBS	7	1.81 **:1.47 *:1.37 *:1.29:1.23:1.19	2.25 **:1.61 *:1.36 *:1.29:1.21:1.18:1.11
RSTS	7	1.21 **:0.88 *:0.75 *:0.72:0.68:0.66	3.48 **:2.90 *:2.63 *:2.36:2.11:1.99:180
GSTS	7	1.70 **:1.06 *:0.79 *:0.72:0.69:0.67	2.55 **:1.56 *:1.38 *:1.29:1.21:1.16:1.09
CBS	7	1.45 **:1.11 *:0.94 *:0.88:0.82:0.79	2.78 **:2.25 *:2.01 *:1.86:1.79:1.71:1.69
Mean	~7	/	/

Note: RBASD: Ratio of Branches of Average Spacing and Distance; RABML: Ratio of the Adjacent Branches’ Mean Length. * and ** Indicates the significant differences in the ratio of the average length of adjacent branch plates at *p* < 0.05 and *p* < 0.01, respectively. The values in front of the above ratio are the means (n ≥ 3) and are calculated based on the measurement data of different plants of the same type.

## Data Availability

All data are contained within the article.
